# Potential of pre-contrast T1 mapping as a marker of interstitial fibrosis in severe aortic stenosis

**DOI:** 10.1186/1532-429X-14-S1-O72

**Published:** 2012-02-01

**Authors:** Andrew Jabbour, Tevfik F Ismail, Callum Ettles, Carl Shakespeare, Sameer Zaman, Oluwatosin Sotubo, Saman S Zaman, Benjamin Hewins, Rick Wage, Ankur Gulati, Pedro F Ferreira, Pierre Croisille, Yanqiu Feng, Raad H Mohiaddin, Taigang He, John Pepper, David N Firmin, Dudley J Pennell, Mario Petrou, Sanjay K Prasad

**Affiliations:** 1 NIHR Cardiovascular Biomedical Research Unit, Royal Brompton and Harefield NHS Foundation Trust, Imperial College London, London, UK; 2 Cardiothoracic Surgery, Royal Brompton and Harefield NHS Foundation Trust, Imperial College London, London, UK; 3 INSERM, Lyon, France

## Background

Aortic stenosis (AS) is associated with increased interstitial myocardial fibrosis (IMF). Although detectable after gadolinium administration by equilibrium-contrast CMR, protocols are lengthy. We hypothesized that pre-contrast T1 mapping, a potential surrogate measure of IMF, would demonstrate reduced T1 in patients with severe AS. We determined the T1 in patients with severe AS and controls before and after contrast, and correlated these values with myocardial strain measures using a high temporal-resolution tagging sequence.

## Methods

A Modified Look-Locker Inversion Recovery (MOLLI) sequence was used to generate eleven T1-weighted images and myocardial T1 derived by fitting a signal intensity-time curve using CMR42®. Short-axis T1 maps were acquired using a 1.5T scanner (Siemens) before and 1,2,5,8,15,20,25 and 30 minutes after contrast. Myocardial tagging images were acquired using single- and multiple-breath-hold CSPAMM sequences in multiple planes and analysed with inTag® (Lyon, France).

## Results

**Figure 1:**
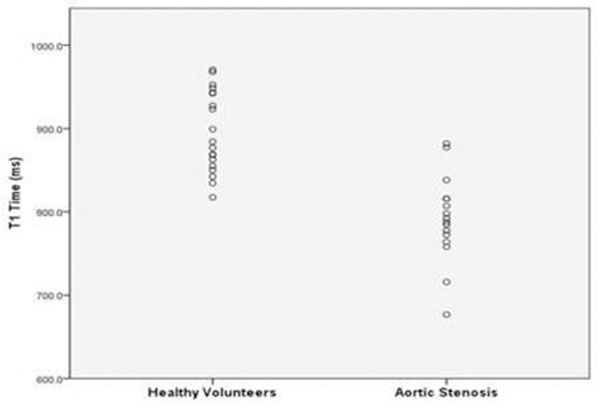


Forty one subjects (age 58 (16), mean (SD); Severe AS, n= 18) were recruited. Subjects with severe AS displayed lower pre-contrast T1 values than healthy controls (791 (52) vs. 896 (49); mean (SD); Fig 1, p<0.001). Pre-contrast T1 correlated well with post-contrast values (e.g. with 15 minute T1, r=0.50, p<0.005); with reduced circumferential max strain (r=0.43, p=0.03), angle δ strain (r=0.78, p=0.001), and motion magnitude peak strain (r=0.87, p<0.001); and with increased septal wall thickness (r=-0.61, p<0.001) and left atrial dilatation (r=-0.55, p=0.001).

## Conclusions

Pre-contrast myocardial T1 mapping demonstrates lower T1 values in patients with severe AS compared to controls and correlates well with both post-contrast T1 and reduced myocardial strain. Further histological validation is required; however, pre-contrast T1 mapping is clinically practical and holds promise for the detection of interstitial fibrosis in severe AS.

## Funding

This project was supported by the NIHR Cardiovascular Biomedical Research Unit of Royal Brompton and Harefield NHS Foundation Trust, the British Heart Foundation, and CORDA. Dr. Jabbour was supported by a Postdoctoral Research Fellowship from the National Health and Medical Research Council of Australia, a Vincent Fairfax Family Foundation Research Fellowship from the Royal Australasian College of Physicians, the St. Vincent’s Clinic Foundation, and the Victor Chang Cardiac Research Institute.

